# Long-Term Effect of Early Post-operative Transcutaneous Electrical Stimulation on Voiding Function After Radical Hysterectomy: A Multicenter, Randomized, Controlled Trial

**DOI:** 10.3389/fmed.2021.677029

**Published:** 2021-09-30

**Authors:** Xiao-wei Li, Lei Gao, Qing Wang, Qiu-bo Lv, Zhi-jun Xia, Hong-wu Wen, Jin-song Han, Yu-mei Wu, Su-mei Wang, Qing Liu, Huan Li, Hai-bo Wang, Yi Li, Shi-yan Wang, Zhi-qi Wang, Xiu-li Sun, Jian-liu Wang

**Affiliations:** ^1^Department of OB/Gyn, Peking University People's Hospital, Beijing, China; ^2^Beijing Key Laboratory of Female Pelvic Floor Disorders, Beijing, China; ^3^Department of OB/Gyn, Beijing Hospital, Beijing, China; ^4^Department of OB/Gyn, Sheng-Jing Hospital of China Medical University, Shenyang, China; ^5^Department of OB/Gyn, Peking University First Hospital, Beijing, China; ^6^Department of OB/Gyn, Peking University Third Hospital, Beijing, China; ^7^Department of OB/Gyn, Beijing Obstetrics and Gynecology Hospital, Capital Medical University, Beijing, China; ^8^Department of OB/Gyn, Beijing Chaoyang Hospital, Capital Medical University, Beijing, China; ^9^Department of OB/Gyn, Gansu Provincial Maternal and Child Health Hospital, Lanzhou, China; ^10^Department of OB/Gyn, Peking University Shen-zhen Hospital, Beijing, China; ^11^Department of Medicine, Peking University Clinical Research Institute, Beijing, China; ^12^Department of Medicine, Peking University Medical Informatics Center, Beijing, China

**Keywords:** pelvic floor electromyography function, examination, quality of life, radical hysterectomy, transcutaneous electrical stimulation, voiding function

## Abstract

**Introduction:** Post-radical-hysterectomy (RH) patients suffer from a series of problems resulting from neurovascular injury, such as bladder dysfunction, which reduce their quality of life. We have designed this study to evaluate the efficacy of transcutaneous electrical stimulation (TENS) on patient rehabilitation after RH for early cervical cancer.

**Materials and methods:** A total of 97 patients were enrolled in a randomized-controlled trial (from January 2015 to December 2019) involving 7 medical centers nationwide. Patients were assigned to either the intervention group (*n* = 46), or the control group (*n* = 51). TENS was given to patients in the intervention group from the 7^th^ day after surgery for a total of 14–21 days. The control group received no TENS. Primary outcomes were measured for residual urine volume and recovery of urination function. Secondary outcomes were measures for urodynamics (UDS), pelvic floor electromyography function examination (PFEmF), and quality of life (QoL).

**Results:** Residual urine volume and improvement in the rate of urination were found to show no significant differences on the 14^th^, 21^st^, and 28^th^ days after surgery. The maximum flow rate (Qmax) in the intervention group was significantly higher than that in the control group on the 28^th^ day, but there were no significant differences in average flow rate, voiding time, time to Qmax, muscle fiber strength, muscle fiber fatigue, and the abnormal rate of A3 reflection on the 28^th^ day and the 3^rd^ mo., as well as in the QoL at 3^rd^ mo., 6^th^ mo., and 12^th^ mo. after surgery.

**Conclusion:** Our study showed no sufficient evidence to prove that TENS under the trialed parameters could improve the subject's voiding function, PFEmF, and QOL after RH. This has provided valuable data for rehabilitation after RH.

**Clinical Trial Registration:** www.ClinicalTrials.gov, identifier: NCT02492542.

## Introduction

Cervical cancer, with almost 0.6 million new cases per year, is globally the 4^th^ most common cancer among women. Approximately 106,000 cases of cervical cancer occurred in China in 2018 (1). Radical hysterectomy (RH), with bilateral pelvic lymphadenectomy, is currently the gold standard surgical treatment for early-stage cervical cancer. It is the definitive therapy, and associated with an excellent prognosis for most patients (2, 3).

However, many patients may suffer from decreased Qol due to the symptoms following RH. It is reported that 70%-85% of cervical cancer patients undergoing RH had *De novo* bladder symptoms (4, 5), 76% of them developed lower urinary tract symptoms (LUTS) in the 12 months after surgery (6). The main reason for post-treatment bladder dysfunction is neurovascular injuries including direct cuts, stretching, and thermal injury. Marloes's (7) study indicated that patients undergoing more radical surgery had more significantly urinary dysfunction.

In clinical practice, providers have tried many methods to prevent and manage urinary retention (UR) in patients undergoing RH, among which post-operative bladder training in the early stages was the most commonly applied. However, Fanfani's randomized trial showed that bladder training did not reduce the rate of UR or re-admission for bladder catheterization (8). Some Chinese doctors reported a potential efficacy when using traditional acupuncture to treat UR after RH (9), but due to limited sample size and poor study design in those studies, more studies are needed to prove its effects.

Electrical stimulation (ES) has been used to treat bladder disorders for many years, especially for UR and incontinence, and its efficacy is promising (10, 11). TENS was the most widely used type of ES. Our previous study (12) showed that TENS could cure acute RH in rats by angiogenesis and nerve fiber regeneration in the detrusor and urethral sphincter, and also increased the expression of collagen. It was reported that ES could promote post-operative recovery of bladder function in prostate cancer (13–17), though there have also been some unsupportive findings (18–21). As the complexity is similar to radical prostatectomy, the same questions also exist in regard to ES treatment for cervical cancer patients undergoing RH.

There are a few studies (22) focused on ES-related post-operative rehabilitation of cervical cancer patients that showed that TENS was effective in preventing UR after RH. Since those studies were not prospective randomized control, we designed this prospective, multi-center, randomized-controlled trial (RCT) to investigate if TENS can improve the voiding function after RH with early cervical cancer.

In this study, UDS was performed post-operatively to evaluate lower urinary tract dysfunction, this method being the gold standard to investigate that particular pathology, while the free flow study was essential in the evaluation of voiding patterns and the characteristics of the emptying phase (23), represented by the Qmax, average flow rate, voiding time, and the time to Qmax. The basic electrophysiological indicators of pelvic floor muscle, which mainly comprise strength, duration, and fatigue of the muscle contraction, were used as an evaluated index for pelvic floor dysfunction (24).

## Materials and Methods

Having been registered at www.clinicaltrials.gov (Identifier: NCT02492542), the RCT was conducted from January 2015 to December 2019. The research protocol was designed based on a methodology published by Xiu-li Sun, etc. in 2017 (25) and reviewed and approved by the ethics boards of the following seven Hospitals: Peking University People's Hospital, Peking University First Hospital, Peking University Third Hospital, Beijing Obstetrics and Gynecology Hospital of Capital Medical University, Beijing Chao-yang Hospital of Capital Medical University, Beijing Hospital, Sheng-Jing Hospital of China Medical University, Peking University Shen-zhen Hospital and Gansu Provincial Maternal and Child Health Hospital.

### Participants and Randomization

Participants of this study were recruited from the nine hospitals. Patients would be enrolled for analysis if they matched the following inclusive criteria: (1) 18–60 years of age; (2) clinically diagnosed as cervical squamous cell carcinoma and staged according to International Federation of Gynecology and Obstetrics Standards [FIGO 2009] as Ia2, Ib1, and IIa1; (3) had underwent RH according to Piver III classification, with a non-nerve-sparing technique; (4) did not have pelvic nodes, margins, and/or the involvement of any vascular/lymphatic spaces; (5) evidenced to have stromal invasion <1/2; (6) histologically graded as 1–2; and (7) willing to provide informed consent. Consenting patients would be excluded if they met any of the following exclusive criteria: (1) had undergone adjuvant chemotherapy/radiotherapy before or after their RH operation; (2) had had nerve-preserving surgery performed; (3) were suspected or confirmed to have urinary system injury; (4) were staged as POP-Q I or higher; (5) had evidenced moderate or severe SUI (Pad test ≥ 10 g); (6) had urinary retention before surgery; (7) experienced severe constipation or difficulty in defecation before surgery; and (8) had investigator-judged uncontrollable epilepsy/central nervous system disease/mental disorder.

Based on statistical calculations, 208 patients must be enrolled to make this study comprehensive enough to achieve a meaningful and representative conclusion. Eligible patients were randomized into the intervention and control groups with a ratio of 1:1 generated by a dynamic randomization system, which was conducted by Peking University Clinical Research Institute, an independent trial administration office. Randomization of the enrolled patients was also conducted with varying block sizes stratified by menopausal status (menopause vs. Non-menopause) and surgical modality (laparoscopic RH or abdominal RH). Participants and investigators were not blinded to the intervention assignments because of the device stimulation and blank control.

However, the study was terminated in June 2019 since the interim analysis on the data of 97 patients showed that the intervention group was not showing significant benefits over the control group, and this data was enough to demonstrate the study hypothesis. It was decided to terminate the study under consideration of the ethical perspective and for the well-being of the research subjects.

A total of 97 patients were enrolled and randomized (46 patients in the intervention group and 51 in the control group) by the termination of the study, of which three withdrew from the study immediately after signing informed consent (1 from the intervention group and 2 from the control group). According to the ITT principle, 94 subjects (45 in the intervention group and 49 in the control group) were included in the final statistical analysis.

### Research Quality Control

Quality control on research procedures was conducted by assuring that: (1) All patients were recruited from gynecological oncology departments and all the principal practitioners conducting the operations were selected senior gynecological oncologists who had been trained for the uniform surgical procedures; (2) follow-up observation was carried out by specialist personnel, who was specifically assigned and trained on the standardized procedure; and (3) Peking University Clinical Research Institute conducted regular supervision.

### Intervention and Control

Patients in the intervention group would receive 30 min TENS twice a day for a total of 14 days (28 sessions) from the 7^th^ day after the operation. The urethral catheter would be removed on the 14^th^ day post the operation. Those who failed to urinate by themselves or had difficulty in fully emptying their bladder (with residual urine more than 100 ml) would receive TENS for additional 7 days.

The TENS parameters used were 1/4/1 Hz of frequency and 270/230/270 μs of pulse width, which were proven to be effective for urinary retention in clinical practices. The intensity of stimulation would be adjusted on a session-by-session basis according to the individual, being applied at the highest threshold that patient could tolerate. The stimulatory electrode was placed on the S3 region and the neutral electrode was placed on the projected position of the bladder on the surface of the body.

Subjects in the control group would receive only routine clinical care during the trial stage. With the exception of the TENS, the patients in the control group would undergo the same study procedures as the intervention group throughout the entire study. The urethral catheter would be removed on the 14^th^ d after the surgery. The catheter would be inserted again and kept for another 7 days if any patient failed to empty their bladder of residual urine. The same procedures would be repeated until the residual urine retention became <100 ml.

### Data Collection and Follow-Up

**Figure 1:**
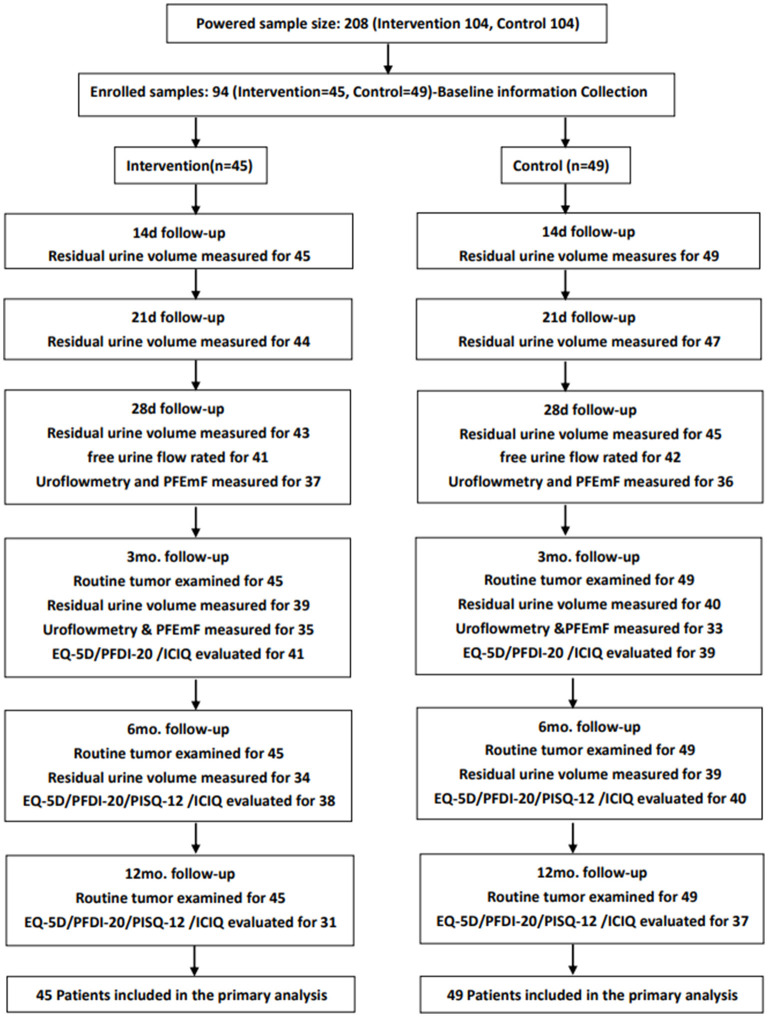
The trial profile of the study: Routine tumor examination, baseline information, gynecological examination (Human Papillomavirus and Thinprep Cytological Testing), tumor markers (squamous cell carcinoma antigen, CA125, CA199, carcinoembryonic antigen), CT or MRI examination, chest radiograph, etc.

Enrolled patients were followed up by the 21^st^ d, 28^th^ d, 3^rd^ mo., 6^th^ mo., 12^th^ mo., 18^th^ mo., and 24^th^ mo. after the operations. Residual urine was evaluated via ultrasound by the 14^th^ d, 21^st^ d, and 28^th^ d post the operation. UDS parameters were assessed by the 28^th^ d and 3^rd^ mo. post-operatively. QoL was evaluated by the European Quality of Life-5 Dimensions (EQ-5D-5L) (26), Prolapse/Urinary Incontinence Sexual Questionnaire (PISQ-12) (27), Pelvic Floor Distress Inventory (PFDI-20) (28), and International Consultations on Incontinence Questionnaire (ICIQ) (29) by the 3^rd^ mo., 6^th^ mo., 12^th^ mo., and 24^th^ mo. after the surgery, except PISQ-12 by the 3^rd^ mo. During the follow-ups, data created from laboratory testing, pelvic examination, chest X-ray examination, and adverse event reports were collected and assessed. The trial profile was shown in Figure 1.

### Study Outcomes

#### Primary Outcomes

The primary outcomes were measured for residual urine volume and recovery of urination function. The recovery of urination function was classified into three levels as: (1) recovered, which refers to the achievement of patient's automatic micturition with residual urine ≤50 ml; (2) improved, which refers to the achievement of patient's automatic micturition with residual urine 50–100 ml; (3) invalidated, which refers to the patient's situation wherein automatic micturition is not achieved, or is achieved but residual urine is ≥100 ml.

#### Secondary Outcomes

The secondary outcomes were evaluated from the following aspects:

(1) UDS parameters: including the Qmax, average flow rate, voiding time, and the time to Qmax.
(2) PFEmF: The strengths and fatigue of the type I and II muscle fibers, the abnormal rate of A3 reflection.
(3) QoL: evaluated by EQ-5D-5L, PISQ-12, PFDI-20, and ICIQ.
(4) Adverse events: skin damage, skin allergies, local pain, etc.

### Statistical Analyses

Statistical analysis was performed using SPSS 19.0. Data are expressed as means ± SD or median ± interquartile. Quantitative data were compared using group *t*-test or Wilcoxon rank-sum test, and qualitative data was analyzed by χ2 test or Fisher exact probability method.

The residual urine volume and the decrease in residual urine volume were evaluated using the Wilcoxon rank-sum test and repeated measures analysis of variance. Wilcoxon rank-sum test and the generalized estimation equation were used to compare the improvement rate of urination, considering the central effect, the Cochran-Mantel-Haenzsel (CMH) test was used to compare the improvement in rate of urination in the 28^th^ d after the operation. UDS parameters, PFEmF, scores of PISQ-12, PFDI-20, and ICIQ were analyzed using the Mann-Whitney *U* test. Scores of EQ-5D-5L were analyzed by Mann-Whitney *U* test and χ2 test or Fisher exact probability method. The level of significance was set to 0.05.

## Results

### Participants Characteristics

In analyzing the data of the 94 enrolled patients, we found no significant differences between the 2 groups in baseline and clinical characteristics (Table 1), indicating that the subjects in the two groups were comparable.

**Table 1:** Baseline and clinical characteristics between the two groups.

		**Intervention**	**Control**	***P***
		**(*N* = 45)**	**(*N* = 49)**	
Age (yr)	<50	26 (57.8)	24 (49.0)	0.39
	≥50	19 (42.2)	25 (51.0)	
Education	< Junior	15 (33.3)	12 (24.5)	0.34
	≥High school	30 (66.7)	37 (75.5)	
Nation	Han	42 (93.3)	43 (87.8)	0.36
	Other	3 (6.7)	6 (12.2)	
Marital status	Married	35 (77.8)	42 (85.7)	0.32
	Divorce/widowed	10 (22.2)	7 (14.3)	
Family cancer history	Yes	3 (6.7)	9 (18.4)	0.09
	No	42 (93.3)	40 (81.6)	
Pregnancy		3.0 ± 2.0	3.0 ± 2.0	0.37
Parity		1.0 ± 1.0	1.0 ± 1.0	0.98
Cesarean section	Yes	14 (31.1)	8 (16.3)	0.09
	No	31 (68.9)	41 (83.7)	
BMI (Kg/m^2^)		23.5 ± 2.8	23.3 ± 2.7	0.69
Operation time (min)		200.0 ± 118.0	184.0 ± 86.0	0.86
Bleeding volume (ml)		200.0 ± 400.0	200.0 ± 300.0	0.59
Surgical approach	Laparoscopy	29 (64.4)	27 (55.1)	0.36
	Trans-abdominal	16 (35.6)	22 (44.9)	

### Lower Urinary Tract Function

#### The Residual Urine Volume

The results of the analysis showed that there was no significant difference in the residual urine volume at the time of each follow-up between the two groups, there was no significant difference between the two groups in the decrease of residual urine volume compared with the baseline either (Table 2). Repeated measures analysis of variance showed that there was no significant difference in the interaction between groups and time (*P* = 0.75).

**Table 2:** The residual urine volume at baseline and by the different periods after intervention.

	**The residual urine volume (ml)**			**Decrease of residual urine volume (ml)**		
	**Intervention (*N* = 45)**	**Control (*N* = 49)**	***P***	**Intervention (*N* = 45)**	**Control (*N* = 49)**	***P***
14^th^ d	68 ± 66	68 ± 72	0.88	–	–	–
21^st^ d	41 ± 37	42 ± 56	0.78	−20 ± 51	−15 ± 53	0.92
28^th^ d	26 ± 45	30 ± 41	0.61	−26 ± 52	−27 ± 50	0.74
3^rd^ mo.	13 ± 22	12 ± 30	0.60	−50 ± 68	−40 ± 75	0.65
6^th^ mo.	10 ± 20	10 ± 32	0.54	−51 ± 63	−47 ± 81	0.69
12^th^ mo.	0 ± 15	7 ± 19	0.36	−51 ± 60	−50 ± 81	0.71

#### The Recovery of Urination Function

There was no significant difference between the two groups in the recovery of urination function as shown in Table 3. The generalized estimation equation showed that there was no significant difference in the interaction between groups and time (*P* = 0.57). There was no significant difference between the two groups in the 28^th^ d by CMH either (*P* = 0.91, OR = 1.249, 95% CI: 0.650, 5.883).

**Table 3:** The recovery of urination function in different periods after intervention.

	**Intervention (*****N*** **= 45)**			**Control (*****N*** **= 49)**			***P***
	**Recovered**	**Improved**	**Invalidated**	**Recovered**	**Improved**	**Invalidated**	
14^th^ d	15 (33.3)	19 (42.2)	11 (24.4)	17 (34.7)	17 (34.7)	15 (30.6)	0.79
21^st^ d	28 (62.2)	12 (26.7)	5 (11.1)	31 (63.3)	11 (22.4)	7 (14.3)	0.98
28^th^ d	29 (64.4)	13 (28.9)	3 (6.7)	35 (71.4)	9 (18.4)	5 (10.2)	0.60
3^rd^ mo.	38 (84.4)	4 (8.9)	3 (6.7)	43 (87.8)	4 (8.2)	2 (4.1)	0.63
6^th^ mo.	39 (86.7)	3 (6.7)	3 (6.7)	44 (89.8)	3 (6.1)	2 (4.1)	0.63
12^th^ mo.	41 (91.1)	2 (4.4)	2 (4.4)	46 (93.9)	2 (4.1)	1 (2.0)	0.61

*The rates of urination improvement of the two groups, which showed the median with interquartile range*.

### UDS Parameters

Although the Qmax of the intervention group was significantly higher than that in the control group by the 28^th^ d (15.7 ± 9.6 vs. 10.45 ± 9.6 ml/s, *P* = 0.02), there was no significant difference between the two groups in the Qmax by the 3^rd^ mo. (19 ± 11.2 vs. 13 ± 8.2 ml/s, *P* = 0.02), as well as in average flow rates (8.1 ± 9.2 vs. 5.55 ± 5.6 ml/s, *P* = 0.07) (9.5 ± 6 vs. 7.7 ± 4.2 ml/s, *P* = 0.22), voiding time (34.05 ± 33 vs. 41 ± 44.9 s, *P* = 0.62) (34.9 ± 27.5 vs. 35.95 ± 30.85 s, *P* = 0.97), and time to Qmax (11.5 ± 13.1 vs. 14.1 ± 22.9 s, *P* = 0.71) (8.1 ± 9 vs. 10.95 ± 12.75 s, *P* = 0.15) by the 28^th^ d and the 3^rd^ mo.

### Pelvic Floor Function on Electromyography

There were no significant differences between the two groups in the strengths of the type I muscle fibers (2 ± 3.5 vs. 3 ± 5, *P* = 1.00) (5 ± 2 vs. 5 ± 4, *P* = 0.99), the strengths of the type II muscle fibers (3 ± 3 vs. 1 ± 5, *P* = 0.16) (−1 ± 2 vs. −1 ± 2, *P* = 0.88), the fatigue of the type I muscle fibers (−1 ± 3.5 vs. −2 ± 3, *P* = 0.93) (−1 ± 2 vs. −1 ± 2, *P* = 0.88), the fatigue of the type II muscle fibers (0 ± 1 vs. 0 ± 1, *P* = 0.80) (0 ± 0 vs. 0 ± 1, *P* = 0.35), as well as the abnormal rate of A3 reflection (13.9 vs. 22.2%, *P* = 0.36) (20.7 vs. 8.3%, *P* = 0.15) by the 28^th^ d and the 3^rd^ mo.

### Quality of Life Questionnaire

There were no statistically significant differences between the two groups in LUTS based on the ICIQ questionnaire (Table 4).

**Table 4:** LUTS based on ICIQ between the 2 groups at 3^rd^ mo., 6^th^ mo., and 12^th^ mo. after operation.

		**3 m**			**6 m**			**12 m**		
		**Intervention**	**Control**	***P***	**Intervention**	**Control**	***P***	**Intervention**	**Control**	***P***
Urinary Urgency	1	32 (94.1 %)	28 (77.8%)	0.18	35 (92.1%)	31 (81.6%)	0.37	26 (92.9%)	27 (75%)	0.40
	2	2 (5.9%)	2 (5.6%)		1 (2.6%)	3 (7.9%)		1 (3.6%)	3 (8.3%)	
	3	0 (0.0%)	2 (5.6%)		0 (0.0%)	1 (2.6%)		0 (0.0%)	1 (2.8%)	
	4	0 (0.0%)	1 (2.8%)		1 (2.6%)	0 (0.0%)		0 (0.0%)	2 (5.6%)	
	5	0 (0.0%)	3 (8.3%)		1 (2.6%)	3 (7.9%)		1 (3.6%)	3 (8.3%)	
Urination pain	1	32 (91.4%)	33 (91.7%)	0.72	37 (97.4%)	35 (92.1%)	0.62	27 (96.4%)	33 (91.7%)	0.63
	2	2 (5.7%)	1 (2.8%)		1 (2.6%)	3 (7.9%)		1 (3.6%)	3 (8.3%)	
	3	1 (2.9%)	2 (5.6%)		0 (0.0%)	0 (0.0%)		0 (0.0%)	0 (0.0%)	
	4	0 (0.0%)	0 (0.0%)		0 (0.0%)	0 (0.0%)		0 (0.0%)	0 (0.0%)	
	5	0 (0.0%)	0 (0.0%)		0 (0.0%)	0 (0.0%)		0 (0.0%)	0 (0.0%)	
Hesitation before urination	1	23 (67.6%)	18 (50.0%)	0.31	27 (71.1%)	21 (56.8%)	0.19	24 (85.7%)	30 (83.3%)	0.93
	2	7 (20.6%)	8 (22.2%)		7 (18.4%)	13 (35.1%)		3 (10.7%)	4 (11.1%)	
	3	3 (8.8%)	4 (11.1%)		2 (5.3%)	0 (0.0%)		0 (0.0%)	0 (0.0%)	
	4	0 (0.0%)	3 (8.3%)		0 (0.0%)	0 (0.0%)		0 (0.0%)	0 (0.0%)	
	5	1 (2.9%)	3 (8.3%)		2 (5.3%)	3 (8.1%)		1 (3.6%)	2 (5.6%)	
Dysuria	1	24 (68.6%)	18 (50.0%)	0.32	28 (73.7%)	25 (65.8%)	0.74	24 (85.7%)	29 (80.6%)	0.61
	2	5 (14.3%)	8 (22.2%)		5 (13.2%)	6 (15.8%)		1 (3.6%)	3 (8.3%)	
	3	0 (0.0%)	2 (5.6%)		1 (2.6%)	3 (7.9%)		0 (0.0%)	1 (2.8%)	
	4	3 (8.6%)	2 (5.6%)		0 (0.0%)	0 (0.0%)		0 (0.0%)	1 (2.8%)	
	5	3 (8.6%)	6 (16.7%)		4 (10.5%)	4 (10.5%)		3 (10.7%)	2 (5.6%)	
Interrupt urination	1	25 (71.4%)	20 (57.1%)	0.57	26 (68.4%)	22 (57.9%)	0.62	24 (85.7%)	26 (72.2%)	0.56
	2	4 (11.4%)	5 (14.3%)		6 (15.8%)	10 (26.3%)		2 (7.1%)	4 (11.1%)	
	3	0 (0.0%)	1 (2.9%)		1 (2.6%)	2 (5.3%)		0 (0.0%)	1 (2.8%)	
	4	0 (0.00%)	1 (2.9%)		1 (2.6%)	0 (0.0%)		0 (0.0%)	0 (0.0%)	
	5	6 (17.1%)	8 (22.9%)		4 (10.5%)	4 (10.5%)		2 (7.1%)	5 (13.9%)	
Urgent incontinence	1	31 (88.6%)	30 (83.3%)	0.78	36 (94.7%)	34 (89.5%)	0.70	23 (82.1%)	30 (83.3%)	0.35
	2	3 (8.6%)	5 (13.9%)		1 (2.6%)	2 (5.3%)		2 (7.1%)	5 (13.9%)	
	3	1 (2.9%)	1 (2.8%)		0 (0.0%)	0 (0.0%)		0 (0.0%)	0 (0.0%)	
	4	0 (0.0%)	0 (0.0%)		0 (0.0%)	0 (0.0%)		2 (7.1%)	0 (0.0%)	
	5	0 (0.0%)	0 (0.0%)		1 (2.6%)	2 (5.3%)		1 (3.6%)	1 (2.8%)	
Stress urinary incontinence	1	29 (82.9%)	30 (83.3%)	0.60	28 (73.7%)	30 (78.9%)	0.22	24 (85.7%)	30 (83.3%)	0.64
	2	5 (14.3%)	5 (13.9%)		4 (10.5%)	6 (15.8%)		2 (7.1%)	4 (11.1%)	
	3	0 (0.0%)	0 (2.8%)		0 (0.0%)	0 (0.0%)		0 (0.0%)	1 (2.8%)	
	4	0 (0.0%)	0 (0.0%)		4 (10.5%)	0 (0.0%)		0 (0.0%)	0 (0.0%)	
	5	1 (2.9%)	0 (0.0%)		2 (5.3%)	2 (5.3%)		2 (7.1%)	1 (2.8%)	

*1, Never; 2, In the past month, such a situation has occurred within less than 10 days; 3, In the past month, such a situation has occurred in 10-20 days; 4, In the past month, such a situation has occurred over 20 days; 5, Available everyday*.

In EQ-5D-5L, state 1 represents no problem, whereas state 5 represents extreme problems. In all dimensions by the 3^rd^ mo., 6^th^ mo., and 12^th^ mo. follow-ups, the percentage of state 1 in the intervention group was higher than that in the control group, except for pain/discomfort by the 3^rd^ mo. and mobility by the 6^th^ mo. The intervention group was significantly better than the control one in usual activities by the 3^rd^ mo. (*P* = 0.02), anxiety/depression by the 12^th^ mo. (*P* = 0.03), and EQ-5D score by the 12^th^ mo. (*P* = 0.01). However, statistical analysis showed no significant difference in the other EQ-5D-5L scores. The PISQ-12 scores and PFDI-20 scores in the intervention group were lower than that in the control one but had no significant difference (Table 5).

**Table 5:** The QoL-questionnaire scores between the two groups at 3^rd^ mo., 6^th^ mo., and 12^th^ mo. after operation.

		**3 m**			**6 m**			**12 m**		
		**Intervention**	**Control**	***P***	**Intervention**	**Control**	***P***	**Intervention**	**Control**	***P***
Mobility	1	37 (90.2%)	33 (84.6%)	0.51	37 (97.4%)	39 (97.5%)	1.0	31 (100%)	36 (97.3%)	0.36
	2	4 (9.8%)	6 (15.4%)		1 (2.6%)	1 (2.5%)		0 (0%)	1 (2.7%)	
	3	0 (0%)	0 (0%)		0 (0%)	0 (0%)		0 (0%)	0 (0%)	
	4	0 (0%)	0 (0%)		0 (0%)	0 (0%)		0 (0%)	0 (0%)	
	5	0 (0%)	0 (0%)		0 (0%)	0 (0%)		0 (0%)	0 (0%)	
Self-care	1	40 (97.6%)	36 (92.3%)	0.35	38 (100%)	37 (92.5%)	0.24	31 (100%)	37 (100%)	–
	2	1 (2.1%)	3 (7.7%)		0 (0%)	3 (7.7%)		0 (0%)	0 (0%)	
	3	0 (0%)	0 (0%)		0 (0%)	0 (0%)		0 (0%)	0 (0%)	
	4	0 (0%)	0 (0%)		0 (0%)	0 (0%)		0 (0%)	0 (0%)	
	5	0 (0%)	0 (0%)		0 (0%)	0 (0%)		0 (0%)	0 (0%)	
Usualactivities	1	39 (95.1%)	30 (76.9%)	0.02	37 (97.4%)	38 (95%)	1.00	31 (100%)	36 (97.3%)	0.36
	2	2 (4.9%)	9 (23.1%)		1 (2.6%)	2 (5%)		0 (0%)	1 (2.7%)	
	3	0 (0%)	0 (0%)		0 (0%)	0 (0%)		0 (0%)	0 (0%)	
	4	0 (0%)	0 (0%)		0 (0%)	0 (0%)		0 (0%)	0 (0%)	
	5	0 (0%)	0 (0%)		0 (0%)	0 (0%)		0 (0%)	0 (0%)	
Pain/discomfort	1	23 (57.5%)	23 (59%)	1.0	30 (78.9%)	24 (60%)	0.15	28 (90.3%)	29 (78.4%)	0.30
	2	16 (40%)	15 (38.5%)		8 (21.1%)	15 (37.5%)		3 (9.7%)	5 (13.5%)	
	3	1 (2.5%)	1 (2.6%)		0 (0%)	1 (2.5%)		0 (0%)	3 (8.1%)	
	4	0 (0%)	0 (0%)		0 (0%)	0 (0%)		0 (0%)	0 (0%)	
	5	0 (0%)	0 (0%)		0 (0%)	0 (0%)		0 (0%)	0 (0%)	
Anxiety/depression	1	29 (70.7%)	26 (66.7%)	0.90	33 (86.8%)	33 (82.5%)	0.60	31 (100%)	30 (81.1%)	0.03
	2	11 (26.8)	12 (30.8%)		5 (13.2%)	7 (17.%5)		0 (0%)	6 (16.2%)	
	3	1 (2.4)	1 (2.6%)		0 (0%)	0 (0%)		0 (0%)	1 (2.7%)	
	4	0 (0%)	0 (0%)		0 (0%)	0 (0%)		0 (0%)	0 (0%)	
	5	0 (0%)	0 (0%)		0 (0%)	0 (0%)		0 (0%)	0 (0%)	
EQ-5D score		0.975 ± 0.11	0.940 ± 0.12	0.32	1.000 ± 0.05	1.000 ± 0.09	0.14	1.000 ± 0.00	1.000 ± 0.06	0.01
EQ-5D VAS		85 ± 13.5	87 ± 15	0.63	90 ± 10	90 ± 15	0.22	95 ± 10	95 ± 11.25	0.37
PISQ-12 scores		–	–	–	8.325 ± 16.5	12.5 ± 17.5	0.22	0 ± 25	12.25 ± 26	0.06
PFDI-20		8.25 ± 19.27	16.59 ± 25.00	0.06	8.29 ± 16.56	12.50 ± 23.75	0.20	0.000 ± 25.00	10.29 ± 26.25	0.08
POPDI-6		0.167 ± 0.333	0.170 ± 0.333	0.39	0.000 ± 0.170	0.167 ± 0.330	0.46	0.000 ± 0.125	0.000 ± 0.167	0.51
CRADI-8		0.000 ± 0.129	0.000 ± 0.250	0.84	0.000 ± 0.000	0.000 ± 0.250	0.12	0.000 ± 0.000	0.000 ± 0.065	0.36
UDI-6		0.167 ± 0.333	0.330 ± 0.500	0.13	0.170 ± 0.333	0.330 ± 0.500	0.38	0.000 ± 0.169	0.167 ± 0.417	0.17

*1, Having no problems; 2, Having slight problems; 3, Having moderate problems; 4, Having severe problems; 5, Unable to*.

*The underline emphasizes P < 0.05*.

## Discussion

Our results demonstrated that TENS could increase the Qmax in the early post-operative period. However, TENS did not make a significant difference in the improvement of residual urine volume and the urination function and did not have an effect on PFEmF or QoL, indicating that TENS cannot work to improve those post-operative problems.

The mean duration of post-operative catheterization varies in different literatures. In Roh's study, the median period for obtaining a PVR volume of <50 ml among patients from the conventional RH group was 18 days, while that in the nerve-sparing RH group was 11 days (30). In another study, 14 days on average was necessary to achieve a PVR volume of <50 ml after nerve-sparing RH (31). In China, the duration of catheterization is usually between 10 and 14 days after the surgery (32). In our research, we removed the catheter 14 days after the operations.

Chuang's research showed that the residual volume of urine tended to increase at 2 and 6 weeks after RH, the mean and maximal flow rates both showed reduction at 2 weeks, 6 weeks, and 3 mos. after surgery (33). This may be attributed to the surgery impairing the parasympathetic motor innervation in maintaining detrusor contractility. In a neuro-urological study, transient neurological changes were observed after RH: pudendal nerve motor latency was prolonged in the 2 and 6 week checks, but returned to baseline levels in the 3 mos (34). Our study also shows that the application of TENS made no significant difference in residual urine volume and the recovery of urination function, although it increased the Qmax by the 28^th^ day. This result suggests that recovery of urination function may be related to the time that has passed allowing for post-operative recovery, which is a natural process, but not to the early TENS.

In Huan Li's study (22), 91 patients diagnosed with stage IA2–IB2 cervical cancer, and treated with RH were enrolled and randomized into two groups. The results showed that low-frequency electrical stimulation was more effective than conventional intervention in preventing urinary retention after RH, and it also intensified the recovery of pelvic muscle strength. A prospective random control trial by Yang (35) demonstrated that sacral and transcranial magnetic stimulation improved pelvic floor dysfunction and QoL of gynecological cancer patients. Hwang's (36) research suggested that transcutaneous electrical stimulation (TES) training resulted in a beneficial effect on sexual function in women with stress urinary incontinence, which was evidenced by the significant differences in pelvic floor muscle strength, power, endurance, and Female Sexual Function Index domain scores in both between-group analyses (TES vs. control group) and within-group analyses (pre-TES vs. post-TSE). Research by Yamanishi (16) showed that electrical stimulation could significantly improve the QOL of patients after radical prostatectomy.

However, Laurienzo (20) reported an unsupportive result based on their research, in which they investigated the effect of electrical stimulation and pelvic floor muscle training on muscle strength, urinary incontinence, and erectile function in men with prostate cancer treated by radical prostatectomy and concluded that the muscle strength recovery occurs independently of the therapy employed; electrical stimulation also did not have an impact on the recovery of urinary continence and erectile function. Marloes (7) found that, although some patients were undergoing more radical surgery, their QoL was not different, suggesting that the surgery itself had few effects on QoL, so the effect of TENS on QoL could not be important. In our study, TENS did not make a significant difference in improvement for residual urine volume and the urination function, and showed no effect on PFEmF or QoL.

Although the exact mechanism of action has yet to be fully understood, TENS was believed to restore the balance between excitation and inhibition in bladder function by modulating the signal traffic to and from the bladder through the sacral plexus. Electrical parameters such as the stimulation frequency, intensity, number, and duration of stimulation sessions were highly variable among studies, which reinforces that there was not a universally established regimen for it. Generally, the frequency of TENS ranges from 2 to 75 Hz (16, 17, 19–21, 37–39). Electrical stimulation with low frequency (2–50 HZ) evoked bladder contraction, resulting in increasing voiding efficiency (39). PFM can be activated with frequencies between 35 and 40 Hz, while the effects at 5–10 Hz spread also to the detrusor muscle (40). Huan Li's study compared the treatment frequency of 35 Hz and 1 Hz, and showed that 1 Hz is more effective (22). The stimulation sites of peripheral nerves were different, such as the sacral nerve roots, tibial nerve, pudendal nerve, and dorsal genital nerves. Electrodes could be placed adjacent to the sacral region (S3), tibial region, clitoral or penile region, or into the vagina or rectum. The sacral or tibial region was likely the most logical site since it directly or indirectly targets the medullar root S3, and the latter was often be preferred as a minimally invasive modality due to the poor tolerability of intra-vaginal and intra-anal electrodes resulting from pain or discomfort (37, 41–43). In this study, frequencies of 1/4/1 Hz was selected, and the electrode site was placed in the S3 region.

The indicators observed in this study are related to urination function, PFM, QoL, etc. While the frequency of TENS for different indicators may be different, so the results may be different if the treatment frequency was changed. The treatment period of this study was 2–3 weeks, also, the results may be changed when the treatment time was extended. TENS parameters need to be explored continuously.

## Conclusions

In summary, our study showed no sufficient evidence to prove that TENS under the trialed parameters could improve the subject's voiding function, pelvic floor muscle strength, and QoL after RH, which has provided valuable data for rehabilitation after RH.

## Data Availability Statement

The raw data supporting the conclusions of this article will be made available by the authors, without undue reservation.

## Ethics Statement

The studies involving human participants were reviewed and approved by 2015PHB050-04. The patients/participants provided their written informed consent to participate in this study.

## Author Contributions

X-lS and J-lW conceived the study and drafted the study design. X-wL, LG, and QW helped with implementation. X-wL wrote the manuscript. J-lW helped to revise the manuscript. H-bW designed the statistical analysis of the study and undertook power calculation. Others are responsible for recruiting patients, performing surgery, collecting data and completing follow-up. YL is responsible for the establishment of medical database. All authors approved the final manuscript.

## Funding

This work was supported by the National Key R&D Program of China (nos. 2018YFC2002204), Clinical Trials.gov (NCT02492542) on June 25, 2015 and Chinese Preventive Medicine Association (nos. 2020-Z-23).

## Conflict of Interest

The authors declare that the research was conducted in the absence of any commercial or financial relationships that could be construed as a potential conflict of interest.

## Publisher's Note

All claims expressed in this article are solely those of the authors and do not necessarily represent those of their affiliated organizations, or those of the publisher, the editors and the reviewers. Any product that may be evaluated in this article, or claim that may be made by its manufacturer, is not guaranteed or endorsed by the publisher.
